# Complex-Forming Properties of the Anti-Inflammatory Sialorphin Derivative Palmitic Acid-Lysine-Lysine-Glutamine-Histidine-Asparagine-Proline-Arginine with Cu(II) Ions in an Aqueous Solution

**DOI:** 10.3390/molecules29010090

**Published:** 2023-12-22

**Authors:** Marek Pająk, Elżbieta Kamysz, Karol Sikora, Jakub Fichna, Magdalena Woźniczka

**Affiliations:** 1 Department of Physical and Biocoordination Chemistry, Medical University of Lodz, Muszyńskiego 1, 90-151 Lodz, Poland; magdalena.wozniczka@umed.lodz.pl; 2 Laboratory of Chemistry of Biological Macromolecules, Department of Molecular Biotechnology, Faculty of Chemistry, University of Gdańsk, 80-308 Gdańsk, Poland; elzbieta.kamysz@ug.edu.pl; 3 Department of Inorganic Chemistry, Faculty of Pharmacy, Medical University of Gdańsk, Al. Gen. J. Hallera 107, 80-416 Gdańsk, Poland; karol.sikora@gumed.edu.pl; 4 Department of Biochemistry, Medical University of Lodz, Mazowiecka 5, 92-215 Lodz, Poland; jakub.fichna@umed.lodz.pl

**Keywords:** sialorphin, Cu(II) complexes, palmitic acid, potentiometry, UV-Vis spectroscopy, protonation constant, stability constant

## Abstract

The present work describes the complexation of the anti-inflammatory sialorphin derivative Pal-Lys-Lys-Gln-His-Asn-Pro-Arg (palmitic acid-lysine-lysine-glutamine-histidine-asparagine-proline-arginine) with Cu(II) ions in an aqueous solution, at a temperature of 25.0 ± 0.1 °C, over the whole pH range. The complexing properties were characterized by potentiometric and UV-Vis spectrophotometric methods. The potentiometric method was used to calculate the logarithms of the overall stability constants (log *β*) and the values of the stepwise dissociation constants (p*K*_a_) of the studied complexes. The percentage of each species formed in an aqueous solution was estimated from the species distribution curve as a function of pH. The absorbance (*A*) and molar absorption coefficient (*ε*) values for the Cu(II)-sialorphin derivative system were determined with UV-Vis spectroscopy. Our studies indicate that the sialorphin derivative forms stable complexes with Cu(II) ions, which may lead to future biological and therapeutic applications.

## 1. Introduction

Sialorphin, which is characterised by the amino acid sequence Gln-His-Asn-Pro-Arg [1,2], is a neuropeptide. It was first identified in the submandibular gland of rats. Its homologue (opiorphin) was later identified in human saliva [3]. It is a natural inhibitor of the neutral endopeptidase (neprilysin, NEP) found on the cell surface. The endogenous opioid peptides enkephalins and atrial natriuretic peptide are the main physiologically relevant substrates for NEP. These mammalian signalling peptides are involved in the control of central and peripheral pain perception, inflammation, arterial tone and mineral homeostasis [4]. Indirect stimulation of opioid receptors by blocking NEP has become a promising pharmacological strategy for the treatment of several diseases and may be more important than the use of classical opioid agonists. In vitro and in vivo studies in animal models have confirmed the efficacy of sialorphin in blocking the activity of NEP. Sialorphin has been shown to exert its anti-inflammatory effect either indirectly, by affecting the levels of enkephalins, or through other mechanisms of action [5]. Sialorphin derivatives have been and are being investigated as potential bioactive materials due to the low bioavailability of sialorphin [6]. They have been studied, among other things, as anti-inflammatory agents and as analgesics with an improved safety profile when compared to conventional opioids [7,8]. For example, some derivatives have been designed to selectively activate the mu opioid receptor while minimising activation of other opioid receptors, which could reduce side effects such as respiratory depression [9].

Palmitic acid is one of the most abundant fatty acids in both animals and plants. It is of great biological importance due to its various roles in physiological processes: it serves as a precursor for the synthesis of other fatty acids, complex lipids and lipid-derived molecules such as sphingolipids and glycerophospholipids [10]; it can modulate inflammatory and immune responses in various tissues [11]; it is a major component of phospholipids and is involved in cell signaling pathways [12]; and it is a principal component of triglycerides, the primary form of energy storage in the body [13]. 

Palmitoylethanolamide (PEA) is a naturally occurring amide of palmitic acid and ethanolamine that reduces pain and inflammation. PEA selectively activates receptor-alpha (PPAR-alpha) in vitro and induces the expression of PPAR-alpha mRNA when applied topically to mouse skin. In two animal models, carrageenan-induced paw edema and phorbol ester-induced ear edema, PEA attenuates inflammation in wild-type mice but has no effect in PPAR-alpha-deficient mice. The above studies indicate that PPAR-alpha mediates the anti-inflammatory effects of PEA and suggest that fatty acid ethanolamide, like its analog oleoiloetanolamide (OEA), may serve as an endogenous ligand for PPAR-alpha [11]. 

Long-chain polyunsaturated fatty acids (PUFAs) are critical structural components of the brain and are essential for normal brain development. The cellular transport and physiological effects of PUFAs are mediated by fatty acid binding proteins (FABPs), which are encoded by the intracellular lipid binding protein gene family. Functional studies have revealed a variety of roles for FABPs in brain development, including neuronal and/or glial cell generation, differentiation, neuronal cell migration and axon patterning. A number of transcription factors have been implicated in the developmental regulation of FABP gene expression in the brain. In addition, FABPs appear to be important downstream effectors of signaling pathways that mediate neuron–glia crosstalk during brain development [12]. Long-chain fatty acids (LCFAs) contain 13 or more carbon atoms. They may be saturated or contain one or more double bonds. Differences in absorption, transport and even destination result from these structural differences. For example, medium-chain fatty acids (MCFAs) are more efficiently absorbed from the gastrointestinal tract than LCFAs and are transported via the portal vein directly to the liver for rapid oxidation. LCFAs are packaged into chylomicrons and travel through the lymphatic system, allowing greater uptake by adipose tissue. Once inside the cell, MCFAs are able to enter the mitochondria without the carnitine shuttle and appear to be preferentially oxidised to fatty acids. LCFAs, on the other hand, require the carnitine shuttle for transport into the mitochondria. When MCFAs replace long-chain triglycerides in the diet, metabolic pathways appear to promote satiety and increase energy expenditure. This may lead to weight control [13]. Myristic acid, palmitic acid and stearic acid are the most common saturated LCFAs in the diet. In their typical dietary sources, there is a lot of overlap. Palm kernel oil, coconut oil and butter are dietary sources of myristic acid. On the other hand, dietary sources of palmitic acid include palm kernel oil, dairy fat, meat, cocoa butter, soybean oil and sunflower oil. For both LDL and HDL cholesterol, myristic and palmitic acids have comparable effects. Overall, they also have little effect on the ratio of total cholesterol to HDL cholesterol [13].

In our case, the addition of a palmitic acid residue (Pal) through a Lys-Lys linker resulted in a compound Pal-Lys-Lys-Gln-His-Asn-Pro-Arg, which was a potent inhibitor of Leu-enkephalin degradation by NEP and significantly attenuated TNBS-induced colitis in mice [14].

Metal ions are important regulators of the activity of many peptides [15,16,17]. Approximately one-third of the known protein structures are metalloproteins, and metal binding or the lack thereof is often implicated in disease, making it necessary to be able to study these systems in detail. Peptide-metal complexes occur naturally, but can also be formed under experimental conditions [18]. It was proved that Cu(II) ions most effectively affect biological functions of peptides. Less effective are metal ions such as Ni(II) and Zn(II). The effect of Cu(II), Ni(II), Zn(II) and their complexes with luliberin (LHRH) on the release of luteinising hormone (LH) and follicle stimulating hormone (FSH) was estimated in in vivo experiments using the method proposed by Ramirez and McCann [19,20]. A metal alone or a mixture of it with LHRH did not affect gonadotropin release at all or no more than LHRH alone. However, the complex of Cu(II) with LHRH resulted in a high release of LH and an even higher release of FSH. This suggests that the copper complex is more effective than metal-free LHRH. The nickel complex showed a similar effect, but to a lesser extent. The zinc complex had similar efficacy to free LHRH, although a higher FSH release was observed. The copper-, nickel- and zinc-LHRH complexes were more potent than the peptide hormone itself and promoted FSH release in the ovariectomized, estradiol and progesterone pretreated rats [20]. The sialorphin Gln-His-Asn-Pro-Arg and its analog, Glp-His-Asn-Pro-Arg, were analyzed in terms of metal binding ability. The analysis of the obtained results shows that both peptides are able to form a series of complexes. However, due to the presence of free N-terminal amino groups, sialorphin is more effective in metal ion binding than its analog. Nevertheless, in basic conditions both peptides involve the amide nitrogen belonging to the side chain of the Asn3 moiety and form a 4N complex with a square planar structure [21,22].

**Figure 1:**
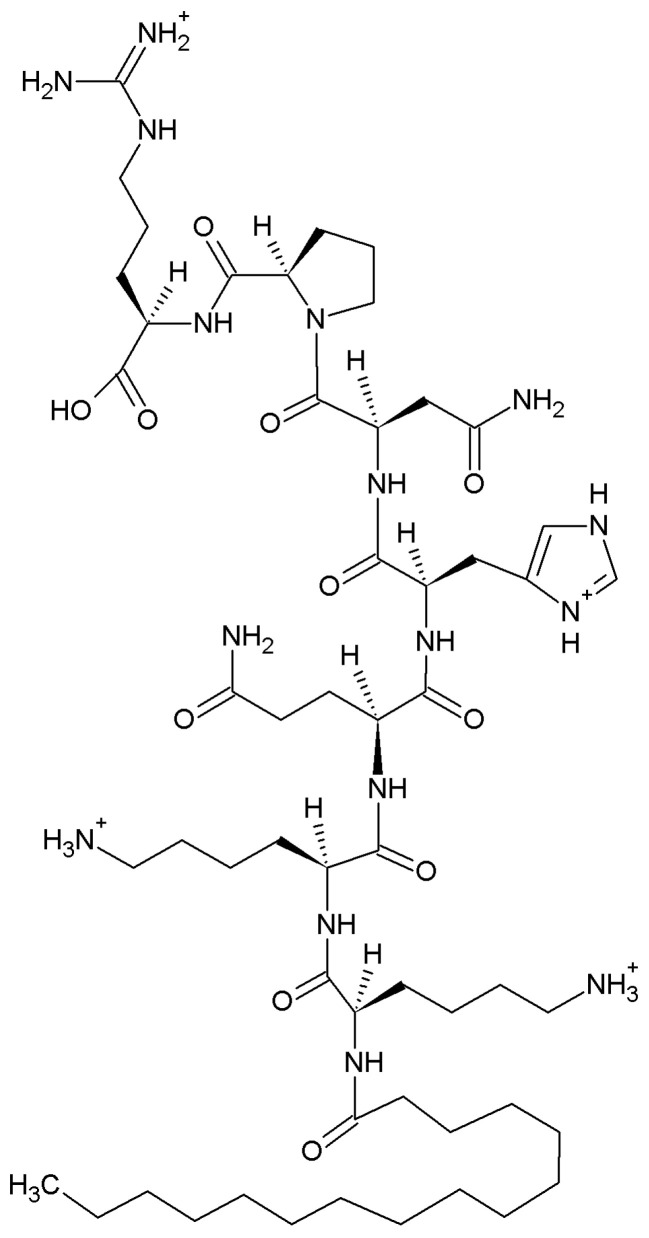
Structure of the protonated sialorphin derivative Pal-Lys-Lys-Gln-His-Asn-Pro-Arg [LH_5_]^4+^.

The aim of this study was to characterize the ability of the sialorphin analog Pal-Lys-Lys-Gln-His-Asn-Pro-Arg (Figure 1) to form complexes with Cu(II) ions in aqueous solution using potentiometric and spectrophotometric techniques. In previous studies, the ligand has shown anti-inflammatory properties in animal models of intestinal inflammation—the results suggest that Pal-Lys-Lys-Gln-His-Asn-Pro-Arg has the potential to become a valuable scaffold for the effective treatment of pain and inflammation, as it may be free of adverse side effects and could become an alternative to current treatments such as non-steroidal anti-inflammatory drugs or corticosteroids [23]. 

## 2. Results and Discussion 

Potentiometric titrations in the tested sialorphin derivative with Cu(II) ions confirmed the formation of six complexes. The calculations are based on previously validated and published results for the determination of the protonation constants of Pal-Lys-Lys-Gln-His-Asn-Pro-Arg using the potentiometric method [24]. The values of the overall stability constants (log_10_ *β_mlh_*) and those of the stepwise dissociation constants (p*K*_a_) of these species are given in Table 1. 

**Figure 2:**
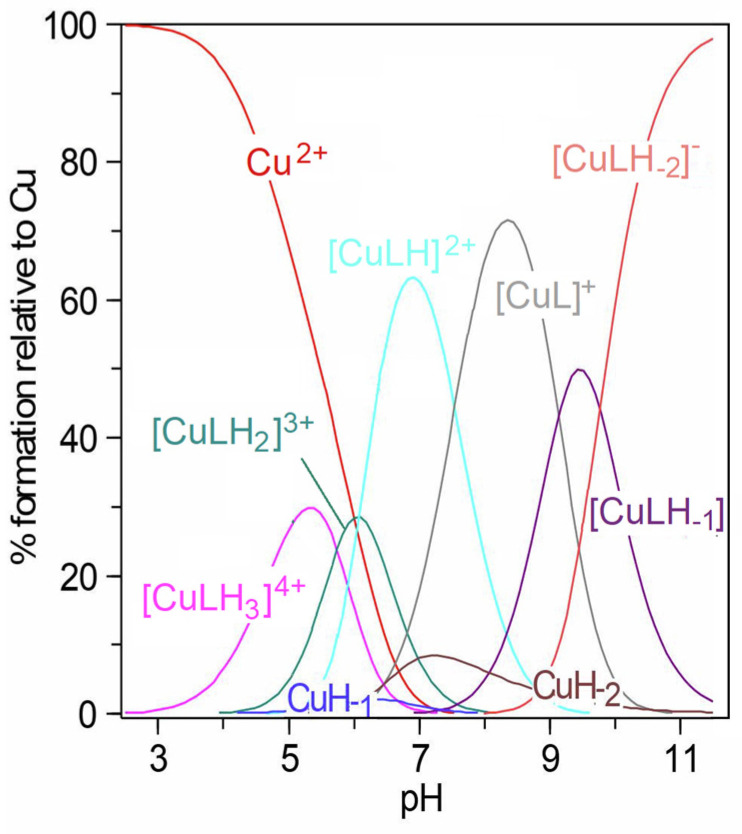
The distribution diagram of species as a function of pH relative to Cu(II), for the complexes formed in the Cu(II)–Pal-Lys-Lys-Gln-His-Asn-Pro-Arg system at ligands: Cu(II) molar ratio 1:1, C_ligand_ = 1.0 × 10^−3^ M obtained from potentiometric data.

Despite the low value of the carboxyl group dissociation constant specified for the ligand itself (p*K*_a1_ = 2.82 [24]), complexation only started above pH 3, as shown in the species distribution diagram (Figure 2). This is influenced by the fact that coordination by carboxyl groups alone is not possible and chelation by the O atom also requires the participation of the nitrogen atom [25,26]. 

**Figure 3:**
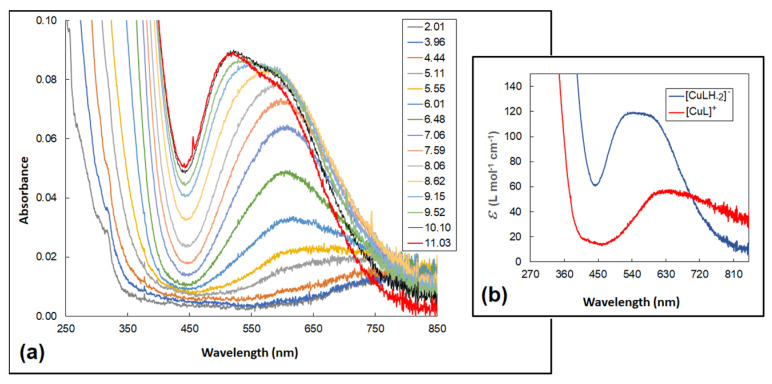
(**a**) UV-Vis absorption spectra of the Cu(II)–Pal-Lys-Lys-Gln-His-Asn-Pro-Arg system within the pH range 2.01–11.03; $C_{{\mathrm{Cu}(\mathrm{ClO}}_{4}{)}_{2}}$ = *C*_ligand_ = 1.0 × 10^−3^ M. (**b**) Molar absorption coefficients (*ε*) of the complexes accepted by HypSpec deconvolution.

**Figure 4:**
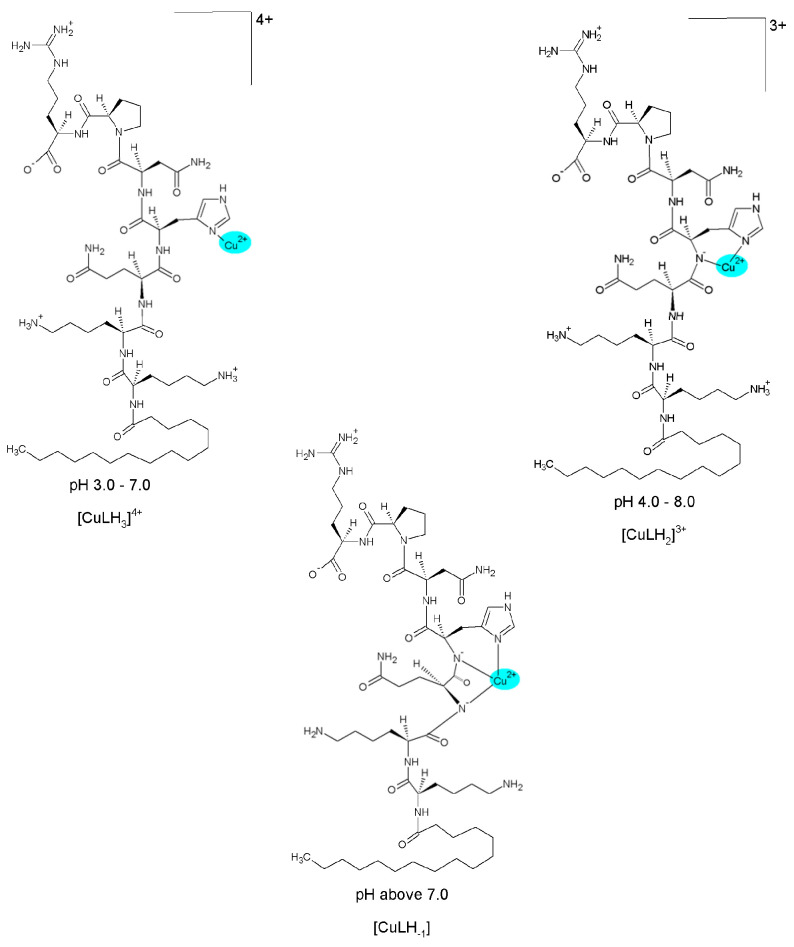
The proposed coordination modes of the complexes in the Cu(II)–Pal-Lys-Lys-Gln-His-Asn-Pro-Arg system in relation to pH.

The first observed complex, [CuLH_3_]^4+^, contained the twice-deprotonated ligand molecule. It can be considered that, in addition to the carboxyl group, the imidazole ring of histidine also dissociated, as the deprotonation constant of the free Pal-Lys-Lys-Gln-His-Asn-Pro-Arg for the imidazole proton was equal to 5.49 [24]. Based on the low value of the related stability constant, $\log_{10}K_{{\mathrm{CuLH}}_{3}}^{\mathrm{Cu}}=3.43$, calculated according to the equation $\log_{10}K_{{\mathrm{CuLH}}_{3}}^{\mathrm{Cu}}=\log_{10}\beta_{113}-\log_{10}\beta_{013}$, where the overall protonation constant for the [LH_3_]^2+^ form was 30.55 [24], it can be assumed that no chelate complex was formed and coordination with the metal ion occurred only through the strongly dative imidazole N3. The formation of complex structures in the Cu(II)–sialorphin system was also confirmed by the electronic absorption spectra (Figure 3a). At a pH of about five, where a maximum concentration of [CuLH_3_]^4+^ was observed, the blue shift of the d-d bands relative to the [Cu(H_2_O)_6_]^2+^ aqua-ion (812 nm, ε = 12) was approximately 100 nm. Previous studies of sialorphin derivatives showed that complexes formed at similar wavelengths contained only one nitrogen donor in the copper coordination sphere [22]. This, therefore, confirms the complexation of the ligand in the [CuLH_3_]^4+^ structure only by the imidazole group (Figure 4). 

Above pH 4, the deprotonation step of the [CuLH_3_]^4+^ allowed the formation of a subsequent complex, [CuLH_2_]^3+^, reaching maximum concentration at pH 6 (Figure 2). The p*K*_a1_ value (Table 1) is similar to that of the deprotonation step of the amide group of another Cu(II) complex [22]. Possible deprotonation of the amide donor and the significant shift of the *d-d* bands (to about 620 nm) indicates that the ligand most likely chelates with metal ions by the {N_imid_,N^−^_amid_} mode (Figure 4). The localization of the *d*-*d* transition at an analogous wavelength range was also confirmed in the case of the planar coordination of the Cu(II) ion by two nitrogen atoms derived from the amine and amide groups of the modified opiorphin [22].

With increasing pH, the formation of two complexes, [CuLH]^2+^ and [CuL]^+^, was found (Figure 2). As the complex [CuL]^+^ is one of the most dominant species in the system, this probably enabled it to be confirmed by HypSpec deconvolution (Figure 3b). However, the formation of [CuLH]^2+^ and [CuL]^+^ did not significantly affect the shift of the electron spectra band. This proved that the only process taking place in the complexes was the dissociation of protons from lysine amine groups. Nitrogen chelation by the imidazole, amide and two amine groups was not possible as it would require the formation of larger than six-membered rings. In aqueous solutions, it was observed that the stability of complexes with a larger ring structure was lower than that of complexes with five- or six-membered rings [27,28]. Additional polynuclear complexes were introduced into the model, in which Cu(II) ions coordinated independently in the {N_imid_,N^−^_amid_} mode and with the lysine amine groups. However, the experimental potentiometric curve did not fit the theoretical curve, which meant that a new model could not be accepted. Bis-ligand complexes were also not confirmed using the Hyperquad 2013 fitting procedure.

The next complex, [CuLH_−1_], was formed at a pH of 7.0 (Figure 2). The blue shift of the *d*-*d* transitions to 531 nm, corresponding to the maximum concentration of the complex at a pH of about 9.5, supported the binding of another nitrogen to the metal ion (Figure 3a). The presence of the isosbestic point at around 600 nm also confirmed that the [CuLH_−1_] structure appears in solutions as a result of the alkalization of the environment. The amide nitrogen of the Gln moiety can be a potential donor for Cu(II) ions, allowing the formation of two chelate rings by {N_imid_, 2N^−^_amid_} coordination (Figure 4). The fourth position was occupied by the water molecule and, as previous studies have shown, alkaline conditions favor proton dissociation from H_2_O [21,22]. However, deprotonation of a coordinated water molecule to produce the hydroxo complex results in a red shift of the absorption maximum, as the OH ion provides a weaker crystal field than the H_2_O ligand [25]. However, the blue shift of the *d*-*d* bands indicates that the dissociation constant p*K*_a4_ corresponds to the amide proton (Table 1).

When increasing the pH to around 11, no significant changes in absorbance were observed; the band shift was 11 nm. However, the isosbestic points appeared at around 508 nm, indicating the formation of a new structure. Kotynia et al. indicated that these spectral properties of the system are attributed to proton dissociation from the guanidyl group of the side chain of the arginine moiety [22]. As shown in the species distribution diagram (Figure 2), the new [CuLH_−2_]^−^ complex reached a concentration maximum within this pH range and appeared as the dominant form in potentiometric titration, which allowed it to be confirmed spectrophotometrically (Figure 3b). The p*K*_a5_ value indicates that the Cu^2+^ ion promoted the deprotonation of the guanidyl group, as the deprotonation constant of free sialorphin corresponding to this proton was higher and equal to 11.88 [24]. However, the structure of the ligand does not allow this moiety to participate in ring formation with the metal ion.

## 3. Materials and Methods

### 3.1. Materials

The ligand Pal-Lys-Lys-Gln-His-Asn-Pro-Arg (palmitoylated at the N-terminus lysyllysylsialorphin Pal-Lys-Lys-sialorphin, where Pal means the residue of hexadecanoic acid) was synthesized manually using the solid-phase method on a 2-chlorotrityl chloride resin (loading 0.3–0.9 mmol/g, 1% DVB, 100–200 mesh, Orpegen Peptide Chemicals GmbH, Heidelberg, Germany) using 9-fluorenylmethoxycarbonyl (Fmoc) chemistry. The peptide’s purity after HPLC purification was higher than 98%, as determined by analytical HPLC. The retention time of the peptide was 11.5 min. in linear gradient from 10 to 80% of 0.1% TFA in acetonitrile and 0.1% TFA in water for 25 min at a flow rate of 1.5 mL/min on a Phenomenex Gemini NX C18 column (4.6 × 150 mm, 110 Å pore size, 5 μm particle size), and UV detection at 214 nm was used. More details on the synthesis and purification of the tested peptide have been described in the literature [23]. 

The mass spectrometry analysis of the synthesized compound was carried out on a matrix-assisted laser desorption/ionization mass spectrometer (MALDI-TOF autoflex maX instrument, Bruker Daltonics, Bremen, Germany) and confirmed the identity of the peptide (M + calc = 1144.74, [M+H] + found = 1145.66). 

Initially, the peptide was obtained as TFA salt. Counter-ions were then exchanged to more biocompatible hydrochlorides (Cl^−^). The exchange to Cl^−^ was performed by lyophilization from 0.05 M HCl solution. The exchange was repeated four times and the final product was lyophilized from deionized water to remove an excess of the acid. Determination of counter-ions and their quantification was performed using ion chromatography (Dionex ICS-5000+, Thermo-Scientific, Waltham, MA, USA) as was described previously [29]. 

The carbonate-free 0.1 M NaOH solution was purchased from J.T. Baker (Radnor, PA, USA). The perchloric acid solution from Laborchemie Apolda (Apolda, Germany) was standardized by titrations with NaOH. The standard solution of sodium perchlorate monohydrate (Laborchemie Apolda, Germany) was used to adjust the ionic medium. Analytical grade Cu(II) perchlorate, Cu(ClO_4_)_2_, was purchased from Chempur, Piekary Śląskie, Poland. Cu(ClO_4_)_2_ solutions were prepared by dissolving an appropriate weight of Cu(II) perchlorate. High-purity argon (Linde, Dublin, Ireland) was used. All solutions were prepared in double-distilled water.

### 3.2. pH-Metric Titrations

Potentiometric titrations were determined by using an automatic titrator system, Titrando 905 (Metrohm, Herisau, Switzerland). A combined glass electrode LL Biotrode (Metrohm, Herisau, Switzerland) was calibrated with NaOH on the hydrogen ion concentration [30]. The solution of the acid was calibrated alkalimetrically and determined with the Gran method [31,32]. The measurements were carried out in a thermostated vessel at a constant temperature of 25.0 ± 0.1 °C and an ionic strength of 0.1 M (NaClO_4_). All titrations were carried out in aqueous solutions in 4 mL samples. A stream of pure argon was passed over the surface of the sample to remove carbon dioxide. 

The system was tested at Pal-Lys-Lys-Gln-His-Asn-Pro-Arg:Cu(II) molar ratio of 1:1 over the entire pH range (2.00–12.00). The total ligand concentration was equal to 1.0 × 10^−3^ M. All experiments were performed in triplicate with very similar reproducibility. The fitting procedure using Hyperquad 2013 software [33] allowed the calculation of the concentration formation constants according to the following formula: *β*_mlh_ = [M*m*L*_l_*H*_h_*]/[M]*^m^*[L]*^l^*[H]*^h^*. The goodness of fit was checked by the objective function *U = Σ_i_*_=1,*m*_ *W_i_ r_i_*^2^, where *W* is the weight, *r* is a residual (equal to the difference between observed and calculated *EMF* values), *m* is the number of experimental points and *n* is the number of refined parameters. The weighting factor *W_i_* is defined as the reciprocal of the estimated variance of the measurements, dependent on the estimated variances of the *EMF* and volume readings. The value of the normalized sum of squared residuals, *δ* = *U*/(*m* − *n*), was compared with the *χ*^2^ (chi-squared) test of randomness at a number of degrees of freedom equal to *m* − *n* [33]. 

Graphical simulations of speciation diagrams based on the calculated constants were generated using HySS 2009. Hyperquad Simulation and Speciation (HySS) is a computer program that provides a system for simulating titration curves and a system for generating speciation diagrams. The calculations are based on equilibria in solution and include the possibility of the formation of a partially soluble precipitate. There are no restrictions on the number of reagents that can be present or the number of complexes that can be formed [34].

### 3.3. Spectrophotometric Measurements

Electronic spectra under argon were recorded on a Cary 50 Bio spectrophotometer equipped with a fiber-optic device (a path length of 1 cm, 5 mm long), (Varian Pty. Ltd., Mulgrave, Australia). This enabled the study of equilibrium systems spectrophotometrically, simultaneously with pH measurements controlled by a Titrando 905 automatic titration (Metrohm, Herisau, Switzerland) kit with a combined polymer microelectrode InLab Semi-Micro (Mettler Toledo, Columbus, OH, USA). Due to the highly disturbing absorption of the nitrate ion at about 300 nm, all the UV experiments were carried out in a perchlorate medium, which was enabled by a combined polymer microelectrode. The ionic strength (I = 0.1 M) was adjusted by NaClO_4_. The electrode was calibrated with buffers at pH 4.00 and 7.00 before use. The fiber-optic probe, 5 mm long and corresponding to a path length of 1 cm, was dipped directly into the thermostated titration vessel (a constant temperature of 25.0 ± 0.1 °C was maintained). A stream of pure argon was passed over the sample surface to obtain oxygen and carbon dioxide solutions freely. After each addition of 0.1 M NaOH carbonate-free and an appropriate time delay to equilibrate the system, the pH and EMF were controlled. The spectrum was recorded with a slow scan (300 nm min^−1^) at selected pH values. 

The tests were first performed for the metal in the absence of the ligand (the total concentration of Cu(ClO_4_)_2_ was equal to 4.92 × 10^−2^ mol·L^−1^). The ligand was then studied in the absence of the metal (the total concentration of the sialorphin derivative was equal to 1.0 × 10^−3^ mol·L^−1^); there was no apparent change in absorbance. The solutions containing Cu(II) ions and Pal-Lys-Lys-Gln-His-Asn-Pro-Arg were prepared in a molar ligand–metal ratio of 1:1 for three samples of this solution (the total metal concentrations were 1.0 × 10^−3^ mol·L^−1^). UV-Vis spectra were recorded in the 200–900 nm range in an aqueous solution. 

## 4. Conclusions

Conventional therapies, which are primarily dependent on ligand administration and its further interaction with specific receptors, are often associated with several side-effects, mainly due to off-target actions related to the PK/PD profile of the drug used. A much more modern concept in disease treatment stems from the inhibition of endogenous enzymes, which affects the levels of natural receptor ligands and allows their finer tuning. The idea has already been translated to clinical conditions as thiorphan, an active metabolite of racecadotril, which has been shown to potently inhibit NEP and has since been used to treat diarrhea, also in irritable bowel syndrome. A natural systemically active regulator of NEP activity, sialorphin, also bears clinical potential, as evidenced in several in vivo examinations of its pharmacological activity against pain and inflammation, among others. However, due to its disadvantageous PK/PD profile, attempts have been made to obtain sialorphin derivatives with more favorable parameters.

Here, we evidenced that the sialorphin analog Pal-Lys-Lys-Gln-His-Asn-Pro-Arg appears to be an effective, specific and promising ligand for Cu(II) ions. The results of potentiometric and spectrophotometric tests indicate that Cu(II) ions can bind to the sialorphin derivative and form complexes through coordination with the functional groups of the relevant amino acid residues of the ligand. The interactions between the amino acid residues can favor a particular peptide conformation, which in turn can have a significant effect on the coordination equilibria between the metal ion and the peptide, both in a thermodynamic and in a structural sense [17,19,20,35,36]. 

It would therefore seem advisable to undertake further experimental studies to demonstrate the role of individual complexes of the equilibrium mixture at physiological pH in biological activity. Such complexes could be investigated in biological systems for their potential to inhibit enzymes and treat pain and inflammation. In the future, sialorphin derivative complexes with Cu(II) may also be tested as bioactive materials, which will warrant further in vitro and in vivo studies.

## Figures and Tables

**Table 1:** Decimal logarithms of the overall formation constants *β_mlh_* = [M*_m_*L*_l_*H*_h_*]/[M]*^m^*[L]*^l^*[H]*^h^* at 25.0 ± 0.1 °C, the stepwise dissociation constants (p*K*_a_) and UV-Vis spectral data. Standard deviations in parentheses after overall stability constants refer to random errors only.

Species	log_10_ *β_mlh_*	p*K*_a_	*λ*_max_ (*ε*_max_)
[CuLH_3_]^4+^	33.98(17)		
[CuLH_2_]^3+^	28.24(15)	p*K*_a1_ 5.74 ^b^	
[CuLH]^2+^	22.16(8)	p*K*_a2_ 6.08 ^c^	
[CuL]^+^	14.62(11)	p*K*_a3_ 7.54 ^d^	619 (54.6)
[CuLH_−1_]	5.49(12)	p*K*_a4_ 9.13 ^e^	
[CuLH_−2_]^−^	−4.28(12)	p*K*_a5_ 9.77 ^f^	537 (118.0)
*σ*; *n* ^a^	0.04; 81		

^a^: *σ*—the value of the normalized sum of squared residuals, *n*—number of titration points. ^b^: p*K*_a1_ = $\log_{10}K_{{\mathrm{CuLH}}_{3}}^{{\mathrm{CuLH}}_{2}}=\log_{10}\beta_{113}-\log_{10}\beta_{112}$. ^c^: p*K*_a2_ = $\log_{10}K_{{\mathrm{CuLH}}_{2}}^{\mathrm{CuLH}}=\log_{10}\beta_{112}-\log_{10}\beta_{111}$. ^d^: p*K*_a3_ = $\log_{10}K_{\mathrm{CuLH}}^{\mathrm{CuL}}=\log_{10}\beta_{111}-\log_{10}\beta_{110}$. ^e^: p*K*_a4_ = $\log_{10}K_{\mathrm{CuL}}^{{\mathrm{CuLH}}_{-1}}=\log_{10}\beta_{110}-\log_{10}\beta_{11-1}$. ^f^: p*K*_a5_ = $\log_{10}K_{{\mathrm{CuLH}}_{-1}}^{{\mathrm{CuLH}}_{-2}}=\log_{10}\beta_{11-1}-\log_{10}\beta_{11-2}$.

## Data Availability

Data are contained within the article.
